# Use of Grading of Recommendations, Assessment, Development, and Evaluation to Combat Fake News: A Case Study of Influenza Vaccination in Pregnancy

**DOI:** 10.2196/10347

**Published:** 2018-11-07

**Authors:** Sidra Zafar, Yacob Habboush, Sary Beidas

**Affiliations:** 1 Department of Internal Medicine Orange Park Medical Center Jacksonville, FL United States

**Keywords:** GRADE, influenza, vaccination, spontaneous abortion, miscarriage

## Abstract

**Background:**

The Grading of Recommendations, Assessment, Development, and Evaluation (GRADE) framework is a validated evaluation tool used to assess the quality of scientific publications. It helps in enhancing clinicians’ decision-making process and supports production of informed healthy policy.

**Objective:**

The purpose of this report was two-fold. First, we reviewed the interpretation of observational studies. The second purpose was to share or provide an example using the GRADE criteria.

**Methods:**

To illustrate the use of the GRADE framework to assess publications, we selected a study evaluating the risk of spontaneous abortion (SAB) after influenza vaccine administration.

**Results:**

Since 2004, the Centers for Disease Control and Prevention and the Advisory Committee on Immunization Practice have recommended influenza vaccination of pregnant women. Previous studies have not found an association between influenza vaccination and SAB. However, in a recent case-control study by Donahue et al, a correlation with SAB in women who received the H1N1 influenza vaccine was identified. For women who received H1N1–containing vaccine in the previous and current influenza season, the adjusted odds ratio (aOR) for SAB was 7.7 (95% CI, 2.2-27.3), while the aOR for women not vaccinated in the previous season but vaccinated in the current season was 1.3 (95% CI, 0.7-2.7).

**Conclusions:**

Our goal is to enable the readers to critique published literature using appropriate evaluation tools such as GRADE.

## Introduction

The Grading of Recommendations, Assessment, Development, and Evaluation (GRADE) framework is a validated evaluation tool used to assess the quality of scientific publications. GRADE was developed by an international group of health professionals, researchers, and guideline developers to standardize the evaluation process of publications [1]. GRADE has been adopted by many organizations including the World Health Organization, American College of Physicians, The Endocrine Society, Infectious Diseases Society of America, The Canadian Task Force on Preventive Health Care, UpToDate, and other domestic and international organizations [2]. The GRADE system helps in enhancing clinicians’ decision-making process and supports the production of informed healthy policy [3].

The present review provides a demonstration of how to use the GRADE system to critique an observational study [4]. It is important to understand the impact of a study on clinical practice, especially if the media reports on the study in a manner that, intentionally or unintentionally, changes the interpretation of the outcomes. The study we selected to demonstrate the use of GRADE was first mentioned in September 2017 in multiple news outlets, such as The Independent and The Washington Post [5]. The paper was published in the journal *Vaccine* under the title of “Association of spontaneous abortion with receipt of inactivated influenza vaccine containing H1N1pdm09 in 2010-11 and 2011-12 [4].” The paper implied a possible link between H1N1 influenza vaccine and spontaneous abortion (SAB) during the first trimester of pregnancy [4]. In response, the Centers for Disease Control and Prevention (CDC) launched a study to address the safety of the H1N1 vaccine in pregnant women. Results from the study will be available in future [6]. The aim of this review was to demonstrate the use of the GRADE framework in evaluating scientific publications to assess their overall quality.

## Methods

We performed a brief review of a recent publication by Donahue et al [4]. The reviewed study is designed as an observational, retrospective, case-control study. Pregnant women with SAB were the targeted population. Eligibility criteria included patients with SAB, diagnosed using clinical examination or ultrasound; age of 18-44 years; known date of last menstrual cycle (LMP); and continuous enrollment with a health care provider for the past 12 months. Subjects with ectopic pregnancy, therapeutic abortion, and history of SAB at less than 5 weeks of gestation were excluded from the study [4].

Cases included 485 women who had SAB and 485 pregnant women in the control group. Both groups were compared to determine whether women with SAB were more likely to have received the 2010-2011 or 2011-2012 seasonal flu vaccine in the proceeding 28 days of SAB. The control cases were selected based on similar characteristics to the cases of SAB, which included maternal age group (<30 or >30 years), approximately similar date of LMP, and enrollment in the same health care plan. Adjustments between the cases and controls were made for smoking during pregnancy, diabetes type 1 or 2, obesity with a body mass index (BMI) of >30, and health care utilization in the prior 1 year. The exposure in this study was receiving the H1N1 influenza vaccine, and the observed outcome was SAB during the first trimester of pregnancy [4]. Vaccine safety data were collected using the Vaccine Safety Datalink (VSD). VSD is a monitoring tool established in 1990 through a collaboration between CDC’s Immunization Safety Office and several integrated health care organizations across the United States [6]. VSD is able to utilize electronic health information from more than 9 million people and abstract information for monitoring and research purposes [6]. The authors of the study abstracted some information from the VSD records such as demographics, vaccination history, and medical outcomes [4].

A regression analysis was performed in the reviewed article; however, the analysis excluded some vital demographics, which may have affected the validity of the results. Some of the demographics are shown in Table 1.

Based on abstracted data from the VSD, the authors calculated an adjusted odds ratio (aOR) of 2.0 (95% CI, 1.1-3.6) for SAB within 1-28 days in both 2010-11 and 2011-12 seasons, comparing vaccinated to unvaccinated women in these seasons. The aORs for 2010-2011 and 2011-2012 were 3.7 and 1.4, respectively, in vaccinated compared with unvaccinated women. On the other hand, the aOR for women who received H1N1–containing vaccine during both the previous (2010-11) and current (2011-12) influenza seasons was 7.7 (95% CI, 2.2-27.3). Meanwhile, the aOR for groups that received the vaccination in 2011-12 but not in 2010-11 was 1.3 (95% CI, 0.7-2.7). When women with previous SAB were excluded, the aOR remained elevated at 6.5 (95% CI, 1.7-24.3); however, the sample size was small, which is represented by the wide CI value (95%, Cl, 2.2-27.3). The study concludes that there is a correlation between SAB and influenza vaccination in the preceding 1-28 days, particularly among women who had been vaccinated in the previous season.

**Table 1:** Major differences in demographics between cases and controls.

Characteristics		Cases, n (%)	Controls, n (%)	*P* value
Age, 35-44 years		157 (32.4)	128 (26.4)	N/A^a^
Body mass index≥30		134 (27.6)	112 (23.1)	N/A
Race, African American		42 (8.7)	20 (4.1)	.008
**Previous spontaneous abortion**				
	≥1	138 (28.5)	125 (25.8)	.32
	≥2	43 (8.9)	26 (5.4)	.03
Smoked during pregnancy		52 (10.7)	34 (7.0)	.05

^a^N/A: not applicable.

## Results

### Analyzing and Interpreting the Study Using Grading of Recommendations, Assessment, Development, and Evaluation

To combat the risks of misinterpreting the reported stories by the news outlets and to standardize the evaluation methods of publications, we recommend using the GRADE system to assess publications. In addition to its validated effectiveness for that purpose, GRADE provides a quantitative evaluation of the evidence [1]. The GRADE tool provides a quantitative score based on the previously mentioned criteria. Table 2 provides the interpretation of the quantitative scores for GRADE. In GRADE, 5 components are evaluated: type of evidence, quality, consistency, directness, and effect size. Next, we assign a value for each component.

### Type of Evidence

Randomized clinical trials are assigned 4 points, while observational studies receive a score of 2 points [1]. The design of case-control studies is based on matching a group of cases to one or more similar control group(s) to compare previous exposures between the groups. Case-control studies use the OR for statistical comparison between the groups [7]. Hence, the design of the study [4] as a case-control study has a score of 2 points.

### Quality

This is a component of the GRADE framework that addresses the methodology and execution of the study by assessing the blinding process, group allocations, follow-ups, and sparse data (missing data). One point is deducted for each problem identified in one of these elements with a maximum deduction of 3 points [1]. The study had 2 quality concerns. First, the 2 groups comprising cases and controls were not appropriately matched given that the authors only matched for age, VSD site, and estimated LMP. Table 1 shows that the case group had more older women aged ≥30 years, a higher BMI of ≥30, more African American women, ≥2 previous SABs, and more smokers during pregnancy [4]. Second, the matched case-control design was problematic as it raised concerns about selection bias due to the lack of appropriate matching characteristics [8]. A preferable design would have been to use a cohort design to evaluate whether pregnant women who did receive the vaccine had a higher risk of SAB. A cohort design would have also been more suitable as the follow-up period is short and all data are available through VSD [8,9]. Hence, we assigned a score of −2 for quality.

### Consistency

This component assesses the consistency of outcomes. A point is deducted for inconsistent results, whereas a point is added for evidence of a dose response, or if adjustment for confounding variables would have increased the effect size [1]. Multiple previous studies have shown consistent results of influenza vaccine having no association with SAB [10-12], which is inconsistent with the conclusion drawn by Donahue et al [4]. Hence, we assigned a score of −1 for this section. Nevertheless, not all inconsistencies between outcomes of studies are “bad.”

### Directness

This component evaluates the issues that may hinder the generalizability of the reported outcomes for the specified population [1]. Table 1 shows that previous SAB was twice as common in the SAB cases as in controls (43/485, 8.9%, vs 26/485, 5.4%; *P*=.03) [4]. The study did not adjust for previous SAB in their adjusted logistic regression models; consequently, this could be a confounding variable that was not accounted for [4]. It also seems quite plausible that women with previous SAB might have had conflicting decisions about whether or not to receive the flu vaccination compared with women who did not. Alternatively, it might be possible that those women could have had SAB regardless of flu vaccination status due to their increased risk for SAB from environmental or genetic risk factors.

Moreover, race was not adjusted for in the study model, even though a significant difference between cases and controls was observed in African American women as shown in Table 1 (*P*=.008). In such observational studies, researchers should always be concerned about whether unmeasured confounding variables might be causing these results [13]. For instance, could socioeconomic status influence the results in such a study? Having comparable groups and adjusting for variables have a direct effect on the internal validity of the study. Also, having a larger representative sample of the population can enhance precision and external validity [7]. Hence, we assigned a score of −1 for this section.

### Effect Size

This component measures the impact of OR, relative risk (RR), or hazard ratio (HR) to provide an estimate of the significance of the results [1]. OR is a measure of association between an exposure and outcome. An OR of 1.0 represents an equal incidence of outcome in both groups, suggesting that the exposure is not a risk factor for that particular outcome. This is referred to as the null value.

**Table 2:** Grading of Recommendations, Assessment, Development, and Evaluation (GRADE) score: quality and interpretation.

GRADE score	Quality	Interpretation
≤1	Very low	Any estimate of effect is highly uncertain
2	Low	Further research is very likely to have an important impact on our confidence in the estimate of effect and is likely to change the estimate
3	Moderate	Further research is likely to have an important impact on our confidence in the estimate of effect and may change the estimate
≥4	High	Further research is very unlikely to change our confidence in the estimate of effect

An OR >1 reflects that the exposed population is more likely to have the observed outcome. An OR <1 means that the exposure is protective. OR is usually presented with a CI wherein the bigger the sample, the smaller the CI. In cases where the CI range crosses the value of 1.0, the OR value will be impaired due to the possibility of having a null, which implies no relationship between exposure and disease [9]. OR in case-control studies should be interpreted with caution due to the nature of the study with only 1 period of observation. Also, the OR equation does not represent the total populations in the exposed and unexposed groups; therefore, it is not possible to directly determine disease rate in such studies [9]. The reviewed study has multiple ORs that are close to the value of 1, which suggests that there is no significant difference between the groups. Hence, we assigned a score of 0 for this section. Our GRADE score for the reviewed study [4] is −2 points (+2 for observational study, −2 for quality, −1 for consistency, −1 for directness, and 0 for effect size). The total score of −2 indicates that the study is of very low evidence.

## Discussion

### Principal Findings

The present review has demonstrated the use of the GRADE framework to quantitively evaluate an observational study, which has been shown to be an effective tool in assessing the study’s strengths and weaknesses. The GRADE framework is easy to use and provides a great estimate of publications’ overall quality. The reviewed study has several limitations; they include the small number of participants, unrepresentative sample, and an observation of an outcome that is rather common during the first trimester, especially between 7th and 12th weeks, of pregnancy [14]. Therefore, the cause of SAB could be multifactorial and not due to the influenza vaccine. Moreover, there are multiple etiologies of SAB including chromosomal abnormalities, intrauterine fetal demise, molar pregnancy, maternal cervical conditions, and hormonal abnormalities. Some of these medical conditions could have made the miscarriage more likely [14].

Another major limitation is the failure to appropriately match case-control groups as the case group had an older population, more SAB, higher BMI, and more smokers during pregnancy. We also need to consider the possibility that some of the pregnant women included in the study might have received influenza vaccination in a nontraditional setting such as in a pharmacy and were not identified as recipients on their medical records [4]. The reviewed article used aOR after adjusting for some variables such as maternal age >30 years, smoking during pregnancy, diabetes type 1 or 2, obesity with a BMI >30, and health care utilization in the prior year. However, lack of adjustment for some other variables such as maternal age ≥35 years and history of SAB, race, and any concurrent infectious illnesses may have significant implications on the aOR as those groups are at higher risk for SAB.

Other issues noted in the study include the possible impact of missing data from the dataset, as 13.6% (66/485) of the data points from the cases and 7.2% (35/485) of the data points from the control group were missing. This is a significant number if we take into consideration the small sample size. Furthermore, some of the outcomes were a result of a post hoc analysis, which refers to an outcome that was not planned for in the study design and was simply noted at a later stage. This is still a major limitation of the study. Physicians should not base their practice on post hoc findings as the results might be flawed due to chance.

A recent survey conducted by the CDC in late 2017 found out that around two-thirds of pregnant women in the influenza season of 2017-18 had not been vaccinated against influenza. Furthermore, only 15.6% of pregnant women who visited a medical provider since July 2017 had received a recommendation for the influenza vaccination, but not offered one; while 25.7% neither received a recommendation nor an offer for the influenza vaccination, 58.7% of pregnant women received a recommendation and an offer to administer the influenza vaccine [15]. It is possible that the effects of mainstream media highlighting the Donahue et al [4] publication might have had an impact on clinical practice as we described.

The CDC’s current recommendation is to vaccinate all pregnant women. It is challenging to convince the public that the reviewed study had various limitations and cannot be generalized to all pregnant women after a media blitz. The media have played a significant part in promoting this study, as multiple news outlets adopted this study’s findings with misleading headlines such as “Miscarriages linked to flu vaccine being administered during pregnancy in new study” from The Independent news agency [5]. We believe information that is preliminary may have potential negative health impact on the general population and might need to be reviewed further before publication.

The media has a very strong impact on the way we think and act as a society [16]. We have yet to recover from the aftermath of a single publication by Wakefield et al [17] that linked autism with measles, mumps, and rubella (MMR) vaccine. Multiple studies were conducted immediately after the publication, and they refuted the proposed link between MMR vaccine and autism [18]. The negative effects from the MMR/autism study created a significant confusion in the community and might have contributed to a number of the measles outbreaks, such as the recent outbreak in Minnesota [19], due to the reluctance of the parents to allow the administration of MMR vaccine to their children [17,18].

As clinicians and researchers, we will be able to facilitate the use of GRADE to analyze the study and its statistical significance. For patients, however, it may not be that easy to find their way through the maze of variables, calculations, and adjusted rates. Therefore, a collaborative discussion with the patient is necessary to explain the overall quality of such publications and recommendations to follow.

### Conclusion

The GRADE framework is a validated tool used to quantitively assess the overall quality of publications. Through the use of GRADE, we uncovered the low-evidence score for the reviewed article. Therefore, the best course of action will be to follow the CDC’s recommendations by providing the influenza vaccine to all pregnant women. Physicians should adopt validated evidence-based tools, such as GRADE, to quantitively assess the overall quality of studies and provide evidence-based practice.

## Abbreviations

aOR: adjusted odds ratio
BMI: body mass index
CDC: Centers for Disease Control and Prevention
GRADE: Grading of Recommendations, Assessment, Development, and Evaluation
HCA: Hospital Corporation of America
HR: hazard ratio
LMP: last menstrual cycle
MMR: measles, mumps, and rubella
RR: relative risk
SAB: spontaneous abortion
VSD: Vaccine Safety Datalink
